# Supplementary material for: Predictors of Hyperuricemia after Kidney Transplantation: Association with Graft Function
Source: Medicina (Kaunas). 2020 Feb 25;56(3):95. doi: 10.3390/medicina56030095 (PMC7143203; doi:10.3390/medicina56030095)
Supplement: Supplementary file 1 [file medicina-56-00095-s001.pdf]

Table S1. HU as a prediction of normal GFR after a three-year follow-up (GFR > 60) *.

Variable	OR	95% CI	*p* value
HU	1.20	0.49; 3.00	0.69
Age	0.95	0.92; 0.99	0.01
Male gender	0.30	0.12; 0.73	< 0.01
BMI ≥ 30	0.17	0.06; 0.54	< 0.01
Hypertension	1.64	0.52; 5.16	0.40
Dyslipidaemia	1.07	0.43; 2.65	0.89
Diabetes mellitus	1.61	0.48; 5.37	0.44
Use of AKE-I/ARB	1.11	0.73; 1.68	0.63
Use of diuretics	0.15	0.06; 0.69	0.02

* Nagelkerke adjusted R^2 = 0.29.
